# Percutaneous CT-guided or endoscopic spinal biopsy with accurate pathogen detection for spinal infection

**DOI:** 10.3389/fneur.2025.1623598

**Published:** 2025-08-22

**Authors:** Jinmei Chen, Qingxin Guo, Luying Tian, Leer Shen, Yi Zhang, Qiong Jiao, Xiaofeng Lian, Xiaohua Chen

**Affiliations:** ^1^Department of Infectious Diseases, Shanghai Sixth People's Hospital Affiliated to Shanghai Jiao Tong University School of Medicine, Shanghai, China; ^2^Department of Pathology, Shanghai Sixth People's Hospital Affiliated to Shanghai Jiao Tong University School of Medicine, Shanghai, China; ^3^Department of Orthopedics, The Sixth People's Hospital Affiliated to Shanghai Jiao Tong University School of Medicine, Shanghai, China

**Keywords:** transforaminal endoscopic spinal biopsy, pathogen, next-generation sequencing, spinal infection, percutaneous CT-guided biopsy

## Abstract

**Aims:**

In this study, we aimed to analyze the pathogen detection results in spinal infections using percutaneous CT-guided biopsy or transforaminal endoscopic spinal biopsy.

**Methods:**

This is a retrospective observational study of patients who underwent biopsy for spinal infection at Shanghai Sixth People’s Hospital between December 2020 and June 2024. Data on demographics, clinical presentations, radiological findings, and histopathology were collected from medical records. Pathogen detection results from different sampling methods were evaluated.

**Results:**

A total of 131 cases of spinal infection were included in the study. The average age of the patients was 59.1 ± 13.8 years. The median time from symptom onset to diagnosis was 2 months [inter-quartile range (IQR): 1 to 4.5 months]. Clinical manifestations included pain (100%) and fever (15.27%), with the lumbar vertebra being the most commonly affected site (110/168, 65.48%). Elevated ESR, CRP, and IL-6 levels were observed in most cases. MR showed high sensitivity (90.90%) but low specificity (2.04%). Pathogen detection was performed using next-generation sequencing (mNGS) and/or microbial culture. Of the 131 patients, 66 underwent percutaneous CT-guided biopsy, and 65 underwent transforaminal endoscopic spinal biopsy. All samples were tested using mNGS, while microbial culture was performed only on abscess samples from CT-guided biopsy cases. The most commonly identified pathogens were *Mycobacterium tuberculosis* (18.9), Staphylococcus (17.9), and Streptococcus (10.5). The positive detection rates using mNGS were 51.52% for percutaneous CT-guided biopsy and 50.77% for transforaminal endoscopic biopsy, whereas the culture-positive rates were 41.67 and 42.86% on abscess samples, respectively. There is no significant difference in the positive rates between the two biopsy techniques (*χ*^2^ = 0.007292, *p* = 0.9319). But the detection rate of Mycobacterium under CT-guided biopsy is higher (18.18% vs. 9.23%).

**Conclusion:**

Accurate pathogen diagnosis is crucial for the diagnosis and treatment of spinal infections. The microbiological detection rates of samples obtained via percutaneous CT-guided are similar to those obtained through transforaminal endoscopic spinal biopsy.

## Introduction

The incidence of spinal infections has been increasing with the overuse of antibiotics and advancements in diagnostic technologies. Spinal infections encompass a variety of conditions, including vertebral osteomyelitis, intervertebral disk inflammation, and epidural abscess (1). The primary cause of spinal infection is bacterial colonization in the spinal region, which spreads from the initial lesion to adjacent segments via the venous and arterial systems. The lumbar spine is the most commonly affected area in vertebral osteomyelitis, where intervertebral space narrowing or vertebral destruction can lead to spinal instability and neurological damage. Spinal infections thus require prompt and active medical management.

Diagnosis mainly depends on clinical presentation, imaging studies and pathogen testing. An accurate diagnosis is crucial for patient management and for ensuring precision medicine, where the right patient receives the right treatment at the right time. Delayed diagnosis may lead to serious complications, including neurological deficits or paraplegia. Timely diagnosis and appropriate management may also reduce the socio-economic burden of spinal infections. The cornerstone of treatment is to identifying the causative pathogen and administering targeted antibiotics. Therefore, this study aims to evaluate the effectiveness of two different sampling methods, percutaneous CT-guided biopsy and transforaminal endoscopic spinal biopsy in pathogen detection.

## Methods

### Patient enrollment

This study was approved by the Ethical Review Committee of the Sixth People’s Hospital of Shanghai. All methods were performed in accordance with the relevant guidelines and regulations. Between December 2020 and June 2024, a total of 131 patients with spinal infections were enrolled from our hospital. The inclusion criteria were as follows: (1) presence of low back pain or spinal pain, and (2) abnormal findings on spinal CT or MR imaging suggestive of a possible spinal infection. All patients underwent spinal biopsy for pathogenic detection.

### Auxiliary laboratory tests

Blood samples were collected for white blood cell count (WBC), erythrocyte sedimentation rate (ESR), C-reactive protein level (CRP), interleukin-6 (IL-6), and other laboratory criteria according to patients’ clinical situations.

### Sample collection and processing

All clinical samples were pre-treatment samples and included serum and pathological tissues. Pathological tissues were collected from the suspected infection sites. Lesion tissue specimens were obtained via percutaneous puncture of the spinal lesions under the guidance of a CT or transforaminal endoscopy. We determined the sample volumes and preparations according to the requirements of each test. All samples were immediately sent for bacterial culture and mNGS based on the manufacturer’s instructions.

### mNGS analysis

DNA was extracted from the clinical specimens using a TIANamp Micro DNA Kit (DP316, TIANGEN BIOTECH) according to the manufacturer’s instructions. Sequencing libraries were constructed and sequenced using an Illumina MiSeq instrument. The reads were analyzed using sequence-based ultra-rapid pathogen identification, which first identifies and subtracts the human host sequences. Microbial genome databases were used to classify the remaining data. The classification reference databases were downloaded from NCBI.^1^ The infectious pathogen was identified using at least 50 unique reads from a single species of bacteria, viruses, fungi, or parasites (2).

### Statistical analysis

Descriptive analysis was used for demographic and clinical characteristics. All the measured data were expressed as the mean ± standard deviation (x ± s). Microbial culture was regarded as the gold standard. Following the extracted data, 2 × 2 contingency tables were derived to determine sensitivity, specificity, positive predictive value (PPV), and negative predictive value (NPV). Sensitivity and specificity were calculated based on the formulas TP (true positive)/(TP + FN) (false negative) and TN (true negative)/(TN + FP) (false positive), respectively. PPV is expressed by the TP/(TP + FP) ratio, while NPV from the TN/(TN + FN). Comparison between two groups was analyzed using *χ*^2^ test or Fisher’s exact test, and a *p* < 0.05 was defined as statistical significance. All statistical analyses were performed using SPSS 20.0 (IBM, Chicago, United States).

## Results

### Demographics and clinical characteristics

The basic characteristics of all 131 patients are summarized in Table 1. The average age of the patients was 59.1 ± 13.8 years. Among them, 78 were males (59.5%) and 53 were female (40.5%). The median time from symptom onset to diagnosis was 2 months (IQR: 1–4.5). The clinical manifestations in 131 cases included with pain in all patients (100%), fever in 20 patients (15.27%), night sweats in nine patients (6.87%), and sepsis in four patients (3.05%). Some patients also presented with neurological symptoms, including lower limb radiative pain in 20 cases, lower limb numbness in 23 cases, limping in 13 cases, dizziness in one case, muscle twitching in two cases, and decreased skin temperature in one case. The infection sites were predominantly located in the lumbar vertebrae (110/168, 65.48%), followed by the thoracic vertebrae (92/168, 17.26%), sacral spine (25/168, 14.88%), and cervical vertebrae (4/168, 2.38%).

**Table 1:** Demographics and clinical characteristics.

Total number	Cases	131
Age	Years, mean (range)	59.1 ± 13.8 (13–86)
Sex	Male (%)	78 (59.5%)
	Female (%)	53 (40.5%)
Diagnosis time	Months, median	2 (IQR,1,4.5)
Symptom	Pain (n, %)	131 (100%)
	Fever (n, %)	20 (15.27%)
	Night sweat (n, %)	9 (6.87%)
	Sepsis (n, %)	4 (3.05%)
	Radiative pain (n, %)	20 (15.27%)
	Lower limb numbness (n, %)	23 (17.56%)
	Limping (n, %)	13 (9.92%)
	Dizziness (n, %)	1 (0.76%)
	Muscle twitches (n, %)	2 (1.53%)
	Low skin temperature (n, %)	1 (0.76%)
Segments of spinal infection	Cervical vertebra (n, %)	4 (2.38%)
	Thoracic vertebrae (n, %)	29 (17.26%)
	Lumbar vertebrae (n, %)	110 (65.48%)
	Sacral vertebrae (n, %)	25 (14.88%)

Among the various predisposing factors identified (Table 2), 25 subjects (19.08%) had received physical therapy, such as acupuncture and moxibustion, prior to diagnosis, 10 subjects (7.63%) were smokers, six subjects (4.58%) were drinkers, and two subjects (1.53%) had a long-time history of hormone use. Additionally, 81 individuals (61.83%) had other underlying conditions, including diabetes (25/131, 19.08%), hypertension (32/131, 24.43%), tumors (4/131, 3.05%), vascular lesions (14/131, 10.69%), nephropathy (2/131, 1.53%), connective tissue diseases (CTD) (9/131, 6.87%), AIDS (1/131, 0.76%), chronic obstructive pulmonary disease (COPD) (1/131, 0.76%), Parkinson’s disease (2/131, 1.53%), cerebral infarction (2/131, 1.53%), liver disease (10/131, 7.63%), leukemia (2/131, 1.53%), rheumatoid arthritis (RA) (2/131, 1.53%), heart disease (5/131, 3.82%), and obesity (1/131, 0.76%).

**Table 2:** Predisposing factors.

Predisposing factors	*n*	%
Physiotherapy	25	19.08%
Smoke	10	7.63%
Drink	6	4.58%
Glucocorticoids	2	1.53%
Diabetes	25	19.08%
Hypertension	32	24.43%
Tumor	4	3.05%
Vascular lesions	14	10.69%
Nephropathy	2	1.53%
CTD	9	6.87%
AIDS	1	0.76%
COPD	1	0.76%
Parkinson	2	1.53%
Cerebral infarction	2	1.53%
Hepatopathy	10	7.63%
Leukemia	2	1.53%
RA	2	1.53%
Cardiopathy	5	3.82%
Obesity	1	0.76%

### Serology and imaging

Serological results showed that the ESR was 61.16 ± 32.87 mm/h, CRP was 26.87 ± 37.87 mg/L, and IL-6 was 18.86 ± 31.36 pg/mL; three were elevated in the majority of patients. The PCT and WBC accounts were elevated in two and eight patients, respectively. Table 3 summarizes the serological values of each patient.

**Table 3:** Laboratory tests.

Laboratory test	
ESR (mm/h)	62.16 ± 32.87
CRP (mg/L)	26.87 ± 37.87
WBC (x10^9/L)	6.49 ± 2.12
Hb (g/L)	119.65 ± 17.21
ALB (g/L)	35.80 ± 4.65
PCT (ng/mL)	0.21 ± 0.94
IL-6 (pg/mL)	18.86 ± 31.36

**Figure 1:**
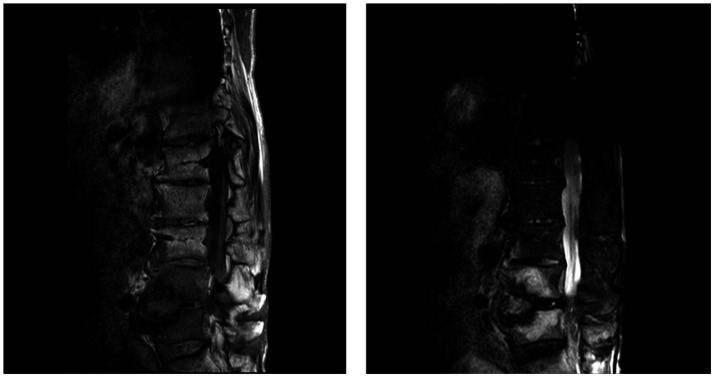
MR images of lumbosacral vertebrae.

Clinical characteristics were assessed, and four diagnostic modalities, namely culture, mNGS, MR and pathology, were used to evaluate 131 patients with spinal infection. Tissue biopsies from infected areas successfully cultured pathogenic bacteria in 33 cases. Using culture as the gold standard, we compared the diagnostic performance of the different methods. mNGS yielded positive results in 67 patients, with a sensitivity of 63.64%, specificity of 53.06%, positive predictive value (PPV) of 31.34%, and negative predictive value (NPV) of 81.25%. Among the 131 patients, 128 had positive MR result, with only three patients (2.29%) showing negative results. MR demonstrated high sensitivity (90.90%) but low specificity (2.04%), with a PPV of 23.81% and NPV of 40%. Pathology (performed on 83 patients) showed sensitivity, specificity, PPV, and NPV of 75.76%, 21.43%, 24.51, and 72.41%, respectively (Table 4 and Figure 1).

**Table 4:** Diagnostic performance of examinations.

Methods	Sensitivity (%, *n*)	Specificity (%, *n*)	PPV (%, *n*)	NPV (%, *n*)
mNGS	63.64%, 21/33	53.06%, 52/98	31.34%, 21/67	81.25%, 52/64
MR	90.91%, 30/33	2.04%, 2/98	23.81%, 30/126	40%, 2/5
pathology	75.76%, 25/33	21.43%, 21/98%	24.51%, 25/102	72.41%, 21/29

PPV, positive predictive value; NPV, negative predictive value.

The L4 and L5 vertebral bodies, corresponding intervertebral disks, and paravertebral signals are abnormal, with uneven enhancement after enhancement. The L5-S1 and L4-L5 intervertebral disks protrude backward with degeneration, while the L3-L4, L2-L3, and L1-L2 intervertebral disks bulge and compress the dural sac. The spinal canal is significantly narrowed, and there is no difference in lumbar spinal signal.

### Spinal biopsy, mNGS, and culture

**Figure 2:**
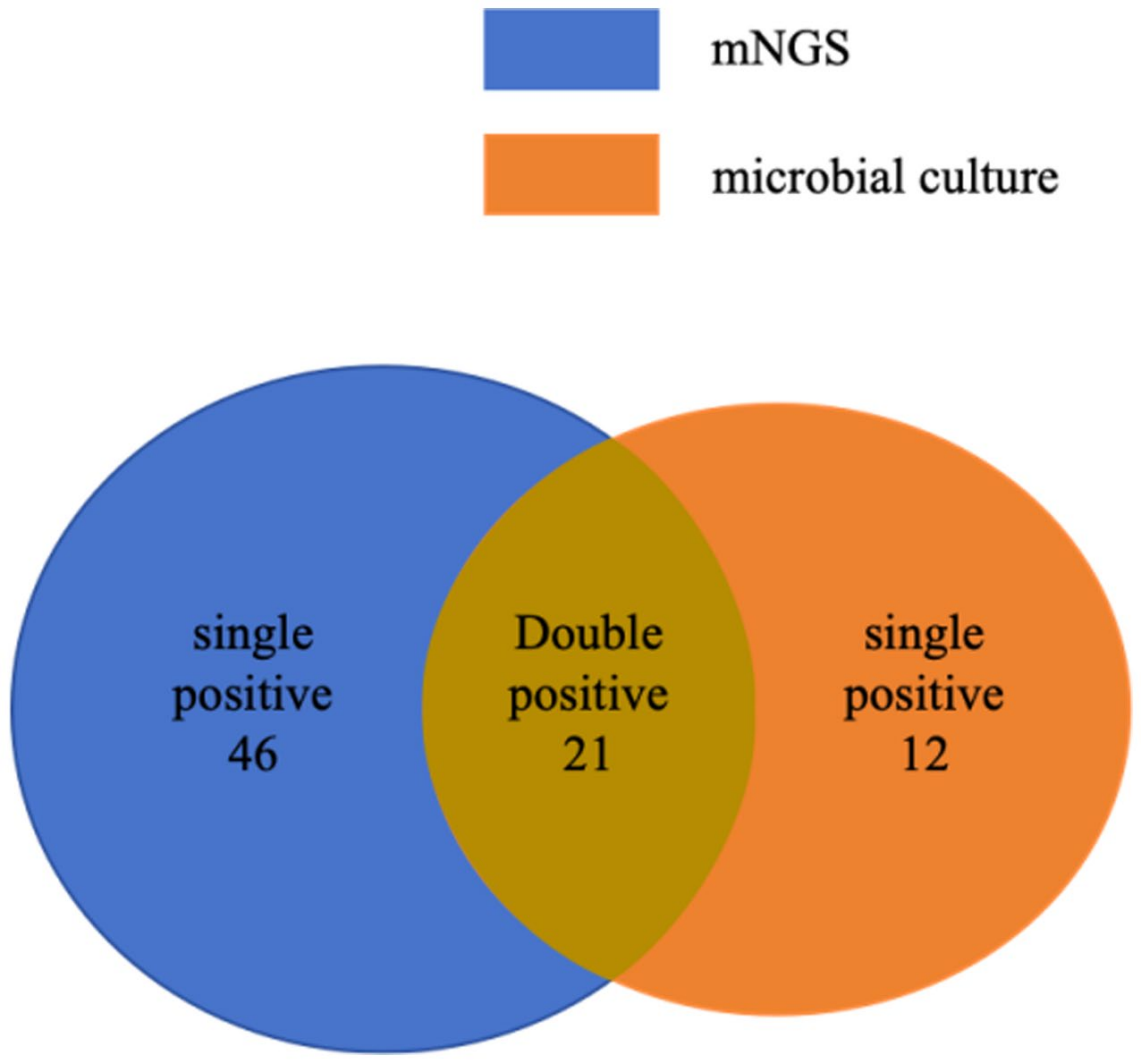
Positive specimen of microbial culture and mNGS.

**Figure 3:**
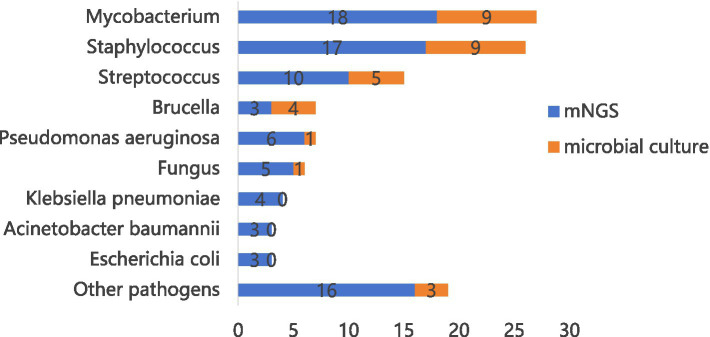
131 patients with detectable pathogenic bacteria.

In this study, to ensure accurate pathogen identification, microbial cultures and mNGS were performed on 131 patients. Of these, 33 patients had positive microbial cultures, and 67 patients had positive mNGS results. Among them, 21 patients were positive for both microbial culture and mNGS (Figure 2). As shown in Figure 3, the most frequently identified bacteria included Mycobacterium (nine cases in microbial culture and 18 cases in mNGS), Staphylococcus (nine cases and 17 cases), followed by Streptococcus (five cases and 10 cases) and *Pseudomonas aeruginosa* (one case and six cases).

All patients underwent needle biopsy, with the samples divided into two groups: 65 samples obtained via transforaminal endoscopic spinal biopsy and 66 samples obtained through percutaneous CT-guided biopsy. The former were examined by pathology, microbial culture, and mNGS, while the later were examined by pathology and mNGS, but microbial culture were conducted only on abscess samples. In the mNGS analysis, Mycobacterium, Staphylococcus, and Streptococcus were the most commonly identified bacteria. The positive rates for transforaminal endoscopic spinal biopsy and percutaneous CT-guided biopsy were 50.77 and 51.52%, respectively (Table 5) (*χ*^2^ = 0.007292, *p* = 0.9319). Of the 26 patients with spinal abscesses, 12 patients underwent transforaminal endoscopic spinal biopsy, while 14 patients underwent. All abscess samples were sent for microbial culture. The bacterial species identified were limited, with Mycobacterium being the most prevalent. There was no significant differences in the positive rate of pathogenic bacteria between endoscopic (41.67%) and percutaneous CT-guided (42.86%) approaches (Table 6) (*p* > 0.9999).

**Table 5:** Pathogenic bacteria by mNGS.

Species	Endoscopic spinal biopsy	CT-guided biopsy	Total
n	65	66	
Mycobacterium	6 (9.23%)	12 (18.18%)	18
Staphylococcus	11 (16.92%)	6 (9.09%)	17
Streptococcus	6 (9.23%)	4 (6.06%)	10
*Pseudomonas aeruginosa*	0 (0)	6 (9.09%)	6
Fungus	1 (1.53%)	4 (6.06%)	5
Brucella	3 (4.62%)	0 (0)	3
*Acinetobacter baumannii*	2 (3.08%)	1 (1.52%)	3
*Escherichia coli*	1 (1.53%)	2 (3.03%)	3
Positive rate	33 (50.77%)	34 (51.52%)	

**Table 6:** Pathogenic bacteria by culture.

Species	Endoscopic spinal biopsy	CT-guided biopsy	Total
n	12	14	
Mycobacterium	4 (33.33%)	1 (7.14%)	5
Streptococcus	1 (8.33%)	1 (7.14%)	2
Brucella	1 (8.33%)	1 (7.14%)	2
Staphylococcus	0 (0)	1 (7.14%)	1
*Enterococcus faecalis*	0 (0)	1 (7.14%)	1
Prevotella intermedius	0 (0)	1 (7.14%)	1
Positive rate	5 (41.67%)	6 (42.86%)	

## Discussion

Spinal infections are relatively rare clinical conditions, and due to their subtle early symptoms, most patients present at a more advanced stage of the disease (3). In our study, the primary reason patients sought medical attention was unexplained pain. The pain is typically confined to the spine, worsened by movement, and may radiate to areas such as the abdomen, buttocks, legs, scrotum, groin, or perineum. Key symptoms in patients with spinal infections include tenderness and muscle spasms in the paraspinal region, along with restricted spinal mobility. Neurological complications, such as spinal cord or nerve root compression and meningitis, can also occur in some patients (4). While most patients experience localized symptoms, a few may develop severe systemic reactions, such as sepsis. The lumbar spine, being the region of the body under the most stress, has a relatively unstable bony structure and significant mobility. This makes it particularly prone to injury, leading to pain and potential neurological issues such as lower limb numbness, sensory deficits, and other functional impairments.

Although early symptoms are often insidious, as the infection progresses, certain blood indicators, such as IL-6, ESR, and CRP, may rise, while WBC and PCT levels generally remain within normal limits. MR is a valuable diagnostic tool in the early stage, helping to delineate the extent of spinal infection. Researches have shown that MR has a high diagnostic value for spinal canal involvement, the severity of endogenous spinal lesions, and the direct involvement of nerve roots and the spine (5). In addition, MR can reveal distinct signal and morphological changes associated with infectious spondylitis of different etiologies. However, while MR shows high sensitivity, it lacks specificity.

The key to diagnosing and treating spinal infections lies in accurately identifying the pathogen and administering the appropriate antibiotic therapy. In our study, pathogens such as Mycobacterium, Staphylococcus, Streptococcus, and *Pseudomonas aeruginosa* were successfully detected, providing a solid etiological foundation for targeted antibiotic treatment. Microbiological specimens were obtained from the affected vertebral bodies and/or intervertebral disk spaces. CT imaging can be used for targeted percutaneous needle biopsy (6). CT-guided biopsy is considered a minimally invasive procedure that allows for precise diagnosis and treatment with a low complication rate. Needle biopsy is a safe, accurate and economical, with diagnostic accuracy ranging from 70 to 100% (7). Tuite (8) noted that intervertebral disk/paravertebral soft tissue biopsy offers superior microbial sensitivity, particularly in cases of atypical infections. For example, the sensitivity of *Mycobacterium tuberculosis* biopsy (62%) is higher than that of purulent organisms (8). In addition, transforaminal endoscopic disk biopsy is also a valuable tool for diagnosing intervertebral disk inflammation. This method has clinical advantages, effectiveness, minimal invasiveness, and a low incidence of complications, making it a safe and efficient treatment option (9). However, it is more costly and associated with greater tissue damage than CT-guided biopsy. In our study, we found no significant differences in pathogenic bacteria detection rate between transforaminal endoscopic spinal biopsy and percutaneous CT-guided biopsy.

The detection of Mycobacterium by mNGS was significantly higher in the CT-guided group compared to the endoscopic group (18.18% vs. 9.23%). This may be attributed to the thoracic spine’s predisposition to tuberculosis (17.26% of cases, Table 1) and the superior suitability of CT guidance for sampling central lesions. *Mycobacterium tuberculosis* spreads to the spine via the bloodstream, particularly colonizing the thoracic spine due to its poor blood supply, which makes it difficult for immune cells to effectively reach the area. Additionally, the thoracic spine is close to the lungs, and when pulmonary tuberculosis occurs, the risk of *Mycobacterium tuberculosis* spreading to the thoracic spine via the bloodstream is higher. Older patients, due to the decline in immune function and the coexistence of chronic diseases (such as chronic obstructive pulmonary disease, and diabetes), are more susceptible to *Mycobacterium tuberculosis* infections. Among older adults, thoracic spine tuberculosis is relatively common in extrapulmonary tuberculosis (17.53%) (10). As a minimally invasive surgical method, transforaminal endoscopy enters the spine through the intervertebral foramen. However, due to limited operating space and restricted vision, it is difficult to precisely locate deep lesions. This is particularly true for deep tuberculosis of the thoracic spine, where the detection rate of *Mycobacterium tuberculosis* may be low. In contrast, CT-guided biopsy can accurately locate the lesion area, making it especially suitable for deep, complex, or hard-to-reach lesions, offering significant advantages for detecting concealed or deep tuberculosis infections.

Identifying pathogenic bacteria is crucial for accurately diagnosing infections. Microbial culture remains the gold standard for infection diagnosis, but it is time-consuming and has a low positive rate and sensitivity. mNGS is an emerging molecular diagnostic tool that can sequences all nucleic acid fragments in a sample (11). mNGS has demonstrated significant potential in pathogen detection and has been found to outperform microbial culture in diagnosing spinal infections (12). With a shorter detection time than culture, mNGS can quickly identify pathogens, detect multiple pathogens in a single test, and even uncover new or rare pathogens (13). For patients with suspected spinal infections, mNGS results offer valuable diagnostic insights and can guide the optimization of antibiotic therapy. Combining mNGS with traditional testing methods significantly enhances pathogen detection rates and improves the effectiveness of treatment (14).

Nevertheless, there are limitations to this study. Firstly, it is a retrospective study with limited data from multiple centers and a small sample size. Additionally, patients without abscesses did not undergo culture sampling during CT-guided biopsy. Lastly, many of the microorganisms identified by mNGS have not been validated through molecular testing.

Clinicians can make a preliminary diagnosis of spinal infection based on clinical features and laboratory tests. The location and extent of lesions are assessed through imaging studies. However, identifying pathogenic bacteria remain the key to determining the infection type and guiding appropriate treatment.

## Data Availability

The original contributions presented in the study are included in the article/supplementary material, further inquiries can be directed to the corresponding authors.

## Footnotes

^1^http://www.ftp.ncbi.nlm.nih.gov/genomes/.
